# Exploring pharmacists' understanding and experience of providing LGBTI healthcare

**DOI:** 10.1016/j.rcsop.2022.100134

**Published:** 2022-04-09

**Authors:** Elizabeth Langdon, Phillip Kavanagh, Mary Bushell

**Affiliations:** a Discipline of Pharmacy, Faculty of Health, University of Canberra, Bruce, Canberra, Australia; b Discipline of Psychology, Faculty of Health, University of Canberra, Bruce, Canberra, Australia

**Keywords:** Gender diversity, Pharmacists, Healthcare, Professional development

## Abstract

**Background:**

Lesbian, gay, bisexual, transgender and intersex (LGBTI) people experience health and wellness challenges additional to and separate from those of the wider population. Extant research has identified that LGBTI patients support education for healthcare providers to improve their access to appropriate care. Community pharmacists have a role in providing appropriate care to LGBTI patients.

**Aims:**

This study explores the experiences of Australian community pharmacists in providing LGBTI healthcare.

**Methods:**

Ten pharmacists were interviewed using a semi-structured interview guide and the major themes were identified using Braun and Clark's thematic analysis.

**Results:**

Analysis revealed a demand for professional education in several areas of LGBTI healthcare, especially gender-affirming hormone replacement therapy and safe communication with this group. Barriers to privacy and confidentiality were revealed, including time constraints, Covid-19 social distancing requirements, and the lack of appropriate consultation rooms. Further there is a need to engage LGBTI patients through greater inclusive advertising.

**Conclusions:**

While there is some improvement, more must be done to promote inclusive pharmacy care for LGBTI people. This study demonstrates a demand from Australian pharmacists for increased professional education on LGBTI related healthcare.

## 1 Background

Community pharmacists are the most accessible health professional. The average Australian visits their pharmacy around 18 times a year. As health professionals, pharmacists are required to provide care in an equitable, patient-centred, and culturally appropriate way to maximise patient health outcomes.^2^^,^^3^ However, to date, it is not known if Australian community pharmacists have sufficient clinical knowledge, etiquette, social understanding and cultural competence to provide equitable and appropriate care for Lesbian, gay, bisexual, transgender and intersex (LGBTI) people.

LGBTI people face health and wellness challenges in addition and separate to those of the wider population, including increased rates of obesity in lesbian and bisexual women,^4^^,^^5^ human immunodeficiency virus (HIV) in men who have sex with men^6^^,^^7^ and high rates of depression, anxiety, and other mood disorders across the scope of LGBTI communities.^8^, ^9^, ^10^, ^11^, 12., 13 There is a multiplication of healthcare disparities for LGBTI people who are also Aboriginal and Torres Strait Islander (Australia's First Peoples).^14^, ^15^, ^16^

Anticipation of discrimination in healthcare settings presents a barrier to improving health outcomes for transgender patients. The Injustice at Every Turn report (IAET),^17^ which examined a survey of over 6000 transgender people in the USA, found that 19% had been refused healthcare by providers due to their transgender status. The report also found that 28% delayed seeking care to avoid discrimination, and 50% reported teaching their providers about transgender healthcare.^17^

A systematic review that explored transgender people's experience of healthcare, found a lack of heath professional knowledge and education across 18 of the 20 included primary studies. ^18^ The review revealed that health professionals had explicitly disclosed to transgender patients their lack of knowledge, and despite best intentions, had a reduced capacity to provide culturally competent and appropriate medical care. Consistent with international findings, a 2018 survey of 928 transgender Australians found strong patient support for more LGBTI-inclusive education for general practitioners (GPs) than is currently being delivered, and a qualitative analysis of the report emphasised the importance of patient-centred care in this population.^9^^,^^19^

A recent study examining the experiences of gender diverse Australians found that when GPs provided care with respect and comfort mental health improved, and when care was provided with discrimination mental health deteriorated. ^20^ To date, there is no study that explores the provision of Australian pharmacist care of gender diverse people.

While there is a paucity of Australian research that has focused on pharmacist and/or LGBTI patient views of healthcare provision, there is a growing body of international literature. Studies have shown that pharmacists have positive attitudes towards caring for transgender people; however, they have both perceived and actual knowledge deficits on how to provide appropriate and culturally competent care.^21^^,^^22^ Transgender patients have also voiced pharmacists should improve their knowledge and awareness on health issues and how to communicate with gender diverse peoples in a safe, positive, and inclusive manner.^20^ Aspects highlighted include misgendering and deadnaming (referring to a transgender or non-binary individual by a name they used before transitioning, e.g., birth name) in Medicare details, improving pharmacist awareness of name and pronoun etiquette may improve healthcare engagement for transgender patients.

A survey of 1701 healthcare students across three universities in the USA found that pharmacy students were among the least clinically prepared of any discipline to care for LGBTI patients, with fewer curricular hours spent on LGBTI healthcare than other disciplines, with this reflected in pharmacy students' low confidence in providing LGBTI healthcare.^23^ To date, there is no research examining Australian pharmacy students' knowledge and skills.

The paucity of research assessing pharmacists' cultural competency in LGBTI healthcare in Australia offers an opportunity to improve the quality of healthcare delivered by the profession, and qualitative analysis of pharmacists' perspectives on LGBTI healthcare permits deep exploration of their experiences as well as their underlying assumptions and attitudes. This study explores community pharmacists' understanding and experiences of providing healthcare to LGBTI patients.

## 2 Methods

### 2.1 Ethics statement

This research gained ethical approval from the Human Research Ethics Committee (HREC) (no. 20219167) in July 2021. All participants provided signed informed consent forms before participating in the interviews. Participants could remove their consent at any time, including after the interview had been conducted. To date, no participants have contacted the research team to remove their consent.

### 2.2 Study design and participant recruitment

A nine-item interview guide was designed to explore participants' experiences across three domains: past learning, current practice, and opportunities for future learning.) Individual questions in the interview guide (see Appendix 1) were aimed at answering the research question and were informed by a narrative literature review. All authors reviewed and agreed upon the final questions. Open-ended questions and a semi-structured approach were employed to increase the depth and detail of responses.^24^

Participants were recruited using purposive sampling to obtain responses from pharmacists in a range of community pharmacy settings. A Facebook post published on the Pharmaceutical Society of Australia's “Early Career Pharmacists” page recruited pharmacists from different geographical areas in Australia (i.e., urban, regional, rural, and remote areas).^25^^,^^26^ Community pharmacies across Australia were also contacted by telephone and direct email based on publicly available information. Invitations to participate in the study were sent to 2158 community pharmacy email addresses. This led to six pharmacists expressing a willingness to participate in the study. The Facebook post led to a further five participants. In total, 11 responses were received from potential participants, leading to 10 interviews conducted and one withdrawal. The audio from one interview failed to record, and the participant was contacted with a written version of the interview guide to provide written responses, which were coded as per the methods section. Thematic analysis was completed using a combination of NVivo qualitative analysis software and manual coding, with validation provided by the co-authors.

Six participants identified as female and four identified as male, no participants identified as another gender. No two participants practiced in the same pharmacy. Participants included early career pharmacists (*n* = 5), established pharmacists, > 10 years of practice, (*n* = 3) and intern pharmacists (*n* = 2). Two participants were pharmacy owners, and one had recently changed workplaces from community to production pharmacy. Seven pharmacists were younger than and three pharmacists were older than 40 years of age. Using the Modified Monash Model criteria, two participants were currently practicing in a rural setting.^27^ No pharmacist described their workplace or home as remote.

### 2.3 Data collection

One-on-one interviews lasting 30–45 min (*M* = 41 mins) were conducted via Zoom remote conferencing software. The interviewer and first author (EL), at the time of the interviews, was completing a Master of Pharmacy degree. There were two other researchers supporting the project, MB a pharmacist and PK a clinical psychologist. It is recognised that in qualitative research it is difficult to avoid personal bias; therefore, information about the research team has been supplied for credibility. Participants answered questions about their eligibility according to the study inclusion criteria, which included registration as an intern or pharmacist and current or recent (last 6 months) employment in an Australian community pharmacy.

### 2.4 Analysis

The audio was manually transcribed by the principal researcher and coded for themes using NVivo software (QSR International, Chadstone, Australia). Data collection and thematic analysis were completed concurrently so findings from early interviews informed later interviews, enabling in-depth exploration of evolving themes. Thematic analysis was conducted using Braun and Clark's six-phase process^28^^,^^29^ and validated by the co-authors. Once consistent themes appeared one further interview was conducted to confirm themes. All authors agreed on the themes. The study was reported according to the Consolidated Criteria for Reporting Qualitative studies (COREQ) checklist.^30^

## 3 Results

### 3.1 Past learning

Two participants, who were recent graduates, reported having LGBTI healthcare related scenarios in tutorials or oral exam practicals, and one of these pharmacists reported having extensive training in HIV and LGBTI related healthcare by an expert in the field as part of their degree structure. The other eight participants described learning nothing about LGBTI healthcare at university:

*“It was very brushed over, no one really mentioned queer people except for the HIV lectures, there was no sexual health education at all.”* Pharmacist 1

*“Dare I say… I think we did one lecture on antiretrovirals, and bits and pieces used for HIV-AIDS treatment, and that was about the extent of things.”* Pharmacist 10

Self-directed study was the most important source of learning for those participants:

*“Most of what I've learned has been kind of reading on my own time from other sources”.* Pharmacist 2

When university courses included material about LGBTI-related healthcare, HIV was the most common topic with other STIs the second closest. These topics were also frequently the first responses of participants when asked what health issues they associated with LGBTI patients. Mental illnesses such as depression and anxiety were the next most named:

*“As a consequence of the path they've been on and the prejudice they've suffered, and the anxiety of coming out and all that stuff… there's quite often a burden of mental health stuff there.”* Pharmacist 3

Awareness of bacterial vaginosis in women who have sex with women (WSW), contraception needs, and the use of non-pharmacological therapeutic goods in binding and tucking for some trans people were mentioned in relation to informal, self-directed learning.

There appeared to be a generation gap between participants who were recent graduates and those who completed university prior to the turn of the millennium, when LGBTI healthcare was taught and documented less thoroughly in formal education settings:

*“But that was before you were born, probably… A lot of things we didn't learn at university in those days, the pharmacy degree is totally different now.”* Pharmacist 9

### 3.2 Current practice

The most common LGBTI-related health services provided by participants in the workplace were dispensing and managing antiretroviral (ARV) medicines, and gender affirming hormone replacement therapy (HRT). One participant described an emerging opportunity to employ a nurse to administer depot injections of ARV medicines:

*“There's an injectable form of an ARV coming to the market soon called Cabenuva and we're looking to perhaps set up a site where nursing staff can come in and administer those doses for patients either during hours or after hours.”* Pharmacist 4

Nicotine replacement therapy (NRT) and other smoking cessation supports were mentioned by another participant.

Although none of the participants were asked about their own LGBTI status, three voluntarily identified themselves as part of the LGBTI community and talked about how their experiences on both sides of the counter. They described the difficulty of experiencing and witnessing homophobia and transphobia at work:

*“I'm used to it at this point, which is the sad part. It is disheartening to see that no one really cares.”* Pharmacist 1

In contrast, pharmacists who were supported to come out to their colleagues described it as a relief.

Pharmacists with a longer duration of experience in the industry, as well as those who described themselves as LGBTI, were more confident in their own ability to provide appropriate care for LGBTI patients than more recent graduates who did not openly identify as LGBTI during the interview.

Transgender healthcare represented a major gap in knowledge for most participants. Confidence in providing counselling for patients undergoing HRT was lower than in providing care for HIV and sexually transmitted infections (STIs) or mental illness. The lack of guidance in professional resources for gender-affirming hormone therapy was identified as a barrier to effective counselling, with one participant stating:

*“I just rely on what the prescription says and hope the doctor or whoever's written it has got it right.”* Pharmacist 5

The lack of dosage protocols in professional resources such as the Australian Medicines Handbook^31^ was cited as a challenge by three participants.

*“Because the robust information [about gender affirming hormones] that you want has been lacking, you can't turn to a page in the AMH and see it sitting there for you, which is where we want to get to. And I think as a society we're getting closer to that.”* Pharmacist 10

Intersex health issues arose as another major health gap, with no participants addressing intersex health during the interviews.

When asked how their workplaces were welcoming and engaging LGBTI patients, most participants responded with “nothing”. Some were upset about this and highlighted the importance of rainbow flag stickers and other visible signs of support; two pharmacists described their workplace making social media posts on significant days like the International Day Against Homophobia, Transphobia and Biphobia as well as displaying visible signs.

“*We've got the ‘welcome here’ signage up and it's all very lovely*.” Pharmacist 10

Two participants emphasised the importance of treating patients with “respect and dignity” regardless of their LGBTI status rather than relying on visible signs.

All but two pharmacists mentioned using search engines when seeking information on LGBTI healthcare. Participants also relied on interprofessional collaboration, and most respondents had a well-developed network of contacts they had built on their own time outside of work. Several participants spoke about the challenge of “starting from scratch” in networking when moving to a new area. The most common service participants referred patients to was sexual health clinics, followed by their general practitioner (GP).

### 3.3 Future learning

When asked about their preferences for learning, participants described a need for a formal training module that could be used by all pharmacy staff (i.e., pharmacists, pharmacy technicians, and retail staff). The benefits of this whole-of-workplace approach would include more proactive intervention, where staff could refer patients to the pharmacist, as well as improving inclusivity in pharmacies. Supplementary materials such as brochures that patients could take home were suggested as helpful aspects of existing training modules on other topics.

All participants explicitly identified gender-affirming hormonal medications as a priority for learning, particularly for transmasculine patients. Dosage protocols, equivalence between different brands and formulations, and information about common adverse effects of HRT were the most common topics specified, followed by information about puberty blockers and how to talk to the parents and caregivers of young HRT patients.

Sexual health, including contraception, STI management, and treating injuries to the genitals, were mentioned by several participants. For example:

*“LGBTI sex education isn't taught in schools, so if a young queer person comes to the pharmacy, it can be difficult to give them that information if the pharmacist doesn't know what to do… I don't think I've ever seen a dental dam in a pharmacy.”* Pharmacist 7

### 3.4 Communication

Some participants who were not part of the LGBTI community described feeling apprehensive when trying to communicate with LGBTI patients, who they perceived as “sensitive”, and one described herself and her colleagues worrying about “saying the wrong thing” or “insulting” patients by accident.

*“How to approach like, find out if their partner is a guy or a girl without asking them if they're a guy or a girl, how do you tease that out of them?”* Pharmacist 6

Other participants noted that being able to communicate safely with LGBTI patients was an important way to build trust with the patients, and improved patient openness, leading to enhanced engagement. “*Once you get past that communication barrier… you disarm some of the [patient's] fear” as their body and condition are no longer “personalised”.*

Pharmacists described the perceptible benefits of establishing rapport with patients using appropriate communication:

*“I have seen their demeanour change when they're like, I can be fully open and honest with you and not get judged”.* Pharmacist 3

*“Some language choices create a green light for being open with your provider.”*

Pharmacist 5

When discussing how pharmacists established rapport with LGBTI patients, all but two mentioned the importance of using gender neutral language particularly when mentioning a patient's partner. Six participants mentioned using the preferred name and pronouns for transgender people and updating their details in dispensing software.

*“It's such a little thing you can do but it's such a big deal to that person.”*

Pharmacist 3

### 3.5 Privacy and confidentiality

When discussing services for LGBTI patients, eight respondents revealed that discretion and privacy were noted as important aspects of providing appropriate pharmacist care. Fear of discrimination, as well as the stigma attached to related health issues such as HIV, were frequently cited as important reasons for heightened concerns about privacy and confidentiality.

Barriers to privacy and confidentiality included time constraints, social distancing requirements during the Covid-19 pandemic, the lack of suitable consultation rooms, and the fast pace of work in community pharmacies, which led to interruptions during private consultations and “neglect” of counselling during the busiest time of day:

*“It's hard when you're trying to help but you can't because there's so much going on, you just can't really do your job as a pharmacist... If it's a new medication we would start a counsel (sic) but because of the social distancing, it's really hard – there's some things we can't just shout across the 1.5 meter [space].”*

Pharmacist 8

There was evidence that patient concerns about privacy added to the urban-rural divide in accessing care. One participant described sending antiretroviral medications by post with discrete packaging from a capital city to rural and regional towns to allow patients to access medication without being recognised by their neighbours and added that some patients were *“a different person in the city than they are at home”.* Pharmacist 9.

## 4 Discussion

While there was a small sample size, we achieved saturation of themes with the final interview revealing no new themes. Consistent with other studies conducted in 2021, recruitment of pharmacist participants was hampered by the Covid-19 pandemic. Pharmacists were serving on the frontline, while continuing to offer essential pharmacy services. Additional cleaning and safety measures were in place to protect staff and consumers, all of which placed an increased demand on workload. Pharmacists managed consumer stockpiling, and were also key to the mass rollout of the Covid-19 vaccine, and routinely experienced staff shortages due to both illness and Covid-19 exposure and subsequent mandatory quarantine. ^32^^,^^33^ This increased workload and reduced the capacity and willingness for pharmacists to volunteer as a research participant.

The limitations notwithstanding, the detail and expertise of the interview responses provide rich qualitative data, which were sufficient to identify several important themes, and will elevate future research and development of professional education. Self-selection bias may be present due to the purposive rather than random sampling design which encouraged participants to opt-in based on personal or professional interest in LGBTI care; but as pharmacists independently choose what continuing professional development (CPD) to complete, this does not necessarily reduce the generalisability of the findings.

There are no current data available on the rate of recruitment and retention of LGBTI staff in the Australian pharmacy industry. The data from this study demonstrates the impact of workplace homophobia and transphobia on participants' motivation, with a potential to cause lost productivity and increase staff turnover. The participants who disclosed their own LGBTI status also had higher confidence in communicating with and providing care for LGBTI patients, which may mean that low retention of LGBTI staff could reinforce poor care quality for patients in community pharmacies where discrimination is frequent. The “confidence gap” between those participants with fewer hours of study and experience and those with more is supported by research on both student and registered pharmacists.^34^,^26^ Short-term improvements in pharmacotherapeutic competency have also been reported after targeted education for pharmacists in the USA.^34^

Gaps in knowledge around intersex and transgender healthcare have been recognised in students and practicing medical professionals^10^^,^^25^, ^26^, ^27^, ^28^, 29., 30, 31., 32, 33, 34, 35., 36, 37 Future studies should explore intersex patients' experiences of healthcare services, including explicitly LGBTI -friendly services.

The importance of visual signs and social signals in welcoming and engaging LGBTI patients is reflected by existing research. An Australian study of young, marginalised people found that gender- and sexuality-diverse youths shared strong preferences for explicitly welcoming health services^38^ and echoed other studies' showing a rainbow flag sticker on a door could improve young LGBTI patients' trust in health services.^39^

Healthcare professionals need to back up symbolism with effective and appropriate services, clinical knowledge, and interpersonal skills to fill patients' needs and provide culturally appropriate care. Pharmacist participants' uncertainty around etiquette and language when speaking to LGBTI patients demonstrates a need for including communication tools in training. Further physical spaces that provide privacy should be utilised or created to promote a welcoming and inclusive service, increasing engagement.

Until recently, little guidance has been available for community pharmacists on etiquette when interacting with transgender patients. A review published in the Australian Journal of Pharmacy in July 2021 includes recommendations such as using gender-neutral language and updating dispensing software to reflect patients' lived reality, as well as the need for a whole-of-workplace approach with training available for all staff.^40^ The recommendations in this review are timely and consistent with concerns and suggestions espoused by participants in this study. This growing body of converging research has the potential to benefit pharmacists, associated professions, LGBTI patients, and the broader health care community in providing an evidence base for areas of ongoing professional development and educational content in university courses.

## 5 Conclusion

This study demonstrates a demand for professional education on LGBTI related healthcare among Australian pharmacists, which is supported by previous research on the needs of LGBTI patients. Pharmacist participants described wanting future training modules to cover gender-affirming hormone therapy, communication skills, and sexual health for LGBTI people, and to be applicable to all roles throughout the community pharmacy. Benefits of such education may include developing pharmacist confidence in providing culturally appropriate care, enhanced patient engagement and retention, and improved health outcomes in the LGBTI community.

## Funding

This research received no specific grant from any funding agency in the public, commercial, or not-for-profit sectors.

## Data availability statement

The dataset presented in this article is available only upon reasonable request since it contains confidential information. Requests to access the dataset should be directed to the corresponding author mary.bushell@canberra.edu.au

## CRediT authorship contribution statement

**Elizabeth Langdon:** Investigation, Methodology, Software, Data curation, Writing – original draft, Formal analysis. **Phillip Kavanagh:** Conceptualization, Methodology, Writing – review & editing, Supervision. **Mary Bushell:** Conceptualization, Methodology, Writing – review & editing, Supervision.

## Declaration of Competing Interest

The authors declare that they have no known competing financial interests or personal relationships that could have appeared to influence the work reported in this paper.

## Appendix 1 Interview guide

1. What health issues come to mind when you think of LGBTI patients?

2. Tell me about what you learned at university about LGBTI health issues and healthcare.

3. What LGBTI-related services do you provide for patients?

4. How confident are you in providing appropriate care for patients in the LGBTI community?

5. What services would you refer a patient to for more information about LGBTI-specific health issues?

6. What does your pharmacy do at the moment to welcome and engage LGBTI patients?

7. What information are you aware of from professional resources about LGBTI healthcare? Where would you go to find out more?

8. What areas of LGBTI healthcare would you like to learn more about?

9. How do you think community pharmacists can contribute to improving health outcomes for LGBTI patients?
